# Are Visitor and Personnel Downtime Restrictions an Effective Biosecurity Measure to Prevent the Indirect Transmission of Pathogens to Livestock?

**DOI:** 10.3390/ani16020205

**Published:** 2026-01-09

**Authors:** Julia Gabrielle Jerab, Evelien Biebaut, Anna Catharina Berge, Ilias Chantziaras, Jeroen Dewulf

**Affiliations:** Department of Internal Medicine, Reproduction and Population Medicine, Faculty of Veterinary Medicine, Ghent University, Salisburylaan 133, 9820 Merelbeke, Belgium; evelien.biebaut@ugent.be (E.B.); cat@bergevetconsulting.com (A.C.B.); ilias.chantziaras@ugent.be (I.C.); jeroen.dewulf@ugent.be (J.D.)

**Keywords:** downtime, animal avoidance period, biosecurity, indirect transmission

## Abstract

Downtime, also known as an animal avoidance period, refers to a 24–72 h period during which individuals who have had contact with one animal species avoid subsequent contact with other susceptible species. This scoping review critically evaluated the effectiveness of downtime as a biosecurity measure. Peer-reviewed studies were identified and analysed using three electronic databases (Pubmed, Web of Science and Scopus). Two experimental studies directly evaluated downtime, nine additional articles examined human nasal carriage of pathogens after exposure to infected animals and seven articles evaluated the effectiveness of hygiene-based biosecurity measures targeting indirect mechanical pathogen transmission via humans (*n* = 14 unique articles in total; topical overlap noted where studies contributed to multiple categories). No evidence indicated an added benefit of downtime over other, more evidence-based measures such as hand hygiene, changing of clothes and boots and showering. While certain studies were able to identify the nasal carriage of pathogens, only one study indicated possible consequent transmission to naïve animals. This limited and species-specific evidence base reveals a critical gap between policy recommendations and scientific support for downtime. Based on these results, no clear benefit or necessity of downtime over other measures have been identified, other than its ability to deter unnecessary visitors, but these do not outweigh the impracticalities associated with an ‘animal avoidance period’. Resources and policy efforts should therefore shift from enforcing downtime toward ensuring and verifying adherence to fundamental hygiene measures.

## Simple Summary

Downtime is a period of 24–72 h during which people who have made contact with one animal species avoid contact with other animals to prevent disease spread. This review evaluated the effectiveness of downtime and found no evidence that downtime provides protection beyond established measures like hand hygiene, changing clothes and boots and showering. Some studies show that humans may carry pathogens in their nasal cavities after contact with infected animals, but only one study indicated possible transmission to other animals under experimental conditions. These findings are limited by the small number of studies, which are primarily pig-focussed and have methodological challenges such as small sample sizes. This review highlights a gap between current biosecurity guidelines recommending downtime and the lack of supporting evidence. While downtime may deter unnecessary farm visitors, it provides no proven benefit over established hygiene measures and may create a false sense of security, leaving animal populations vulnerable. Resources and policy should therefore focus on implementing and verifying core hygiene measures rather than enforcing downtime.

## 1. Introduction

In 1967 and 1968, the United Kingdom (UK) experienced a foot and mouth disease (FMD) epidemic which resulted in 2364 infected farms [1]. Two years later, a study by Sellers et al. [2] found that FMD virus (FMDV) could be recovered from the nose, throat and saliva of one of eight people who made contact with infected animals, up to 28 h after exposure. In a follow-up experimental challenge study, FMDV was transmitted to four steers by people exposed to infected pigs, after they coughed and sneezed into the muzzle of the steers for two and a half minutes [3]. These findings were interpreted as evidence that recently exposed individuals might introduce FMDV to naïve herds via viruses present in the upper respiratory tract, and they catalysed the introduction of downtime as a temporal separation between successive animal contacts. Downtime is defined as an animal avoidance period of 24 to 72 h in which individuals avoid contact with other animals after exposure to susceptible animals which are potentially infected.

External biosecurity measures, including downtime, aim to reduce the risk of pathogen introduction into a herd [4]. While the largest risk of pathogen introduction is the introduction of new animals [5], personnel and visitors have also been shown to form a significant risk of disease introduction [6,7,8], mostly acting as mechanical vectors through contaminated clothes, skin, boots and hair [5]. For a limited number of zoonotic pathogens, such as *Mycobacterium tuberculosis*, *Brucella* spp. and *Salmonella enterica*, humans can also act as biological vectors when they themselves are infected and are actively shedding a pathogen [9]. As such, downtime has been implemented as a biosecurity measure to help minimise the risk of people as a source of pathogen introduction and spread into a herd or farm [4,10,11,12].

Downtime is mostly implemented in pig and poultry production. Pig farms often require a downtime of 24 to 48 h for the prevention of the spread of pathogens such as the porcine reproductive and respiratory syndrome virus (PRRSV), and in poultry farms, the measure is mostly implemented to prevent the introduction of avian influenza (AI) virus [5,13]. However, its implementation varies by region and farm type. Biocheck.UGent surveys show that 19% of surveyed intensive pig farms (*n* = 40195) and 18–30% of poultry farms (*n* = 7282; *n* = 1833) required a downtime period of at least 12 h [13].

Legislation and guidelines on biosecurity measures, which include downtime, are dispensed through different channels, in different forms and with varying areas of applicability. Globally, the World Organisation for Animal Health (WOAH) recommends that all poultry establishments ensure that all ‘personnel and visitors should not have had any recent contact with other poultry, poultry waste, or poultry processing plant(s)’ [14]. Within the European Union (EU), downtime is included as a measure for certain listed diseases [15,16]. National governments have included downtime in their own legislation, such as travel restrictions for travellers entering the United States of America or Australia who have come into contact with livestock before travel [17,18]. Biebaut et al. [19] found that downtime restrictions were included in the national legislation in 3 of 24 surveyed countries. Industry-specific guidelines have also emerged, often linked to outbreaks, such as the Netherland’s hygiene protocol for poultry farms requiring visitors to avoid other poultry holdings for at least 24 h [20]. Supplementary Table S1 provides an overview of legislation and other guidelines including downtime as a biosecurity measure.

Despite its widespread application and inclusion in legislation and guidelines, the scientific foundation of downtime has been questioned. The original studies by Sellers et al. [2,3] relied on extraordinary measures to transmit the pathogen and did not confirm whether viruses recovered from human nasal samples were infectious under normal farm conditions [21]. In addition, there is a marked scarcity of species-specific data for ruminants and poultry, even though downtime is routinely recommended for these production systems. With limited recent data and significant practical consequences for personnel and professionals such as veterinarians and catching crews, the necessity and effectiveness of downtime remain unclear.

While biosecurity is associated with better animal health and reduced antimicrobial use [22,23,24], the prioritisation of measures without robust evidence may lead to a false sense of security, negatively impacting effective on-farm biosecurity.

To address this, a scoping review was conducted to critically evaluate the effectiveness of downtime as a biosecurity measure based on the available literature, with the following research question: ‘Is there evidence that downtime is an effective biosecurity measure to prevent the indirect transmission of pathogens from people to livestock?’

## 2. Materials and Methods

A literature search using three electronic databases (Web of Science, PubMed and Scopus) was conducted between 5 August 2023 and 22 December 2023. The scoping review procedure was conducted following those established by the Preferred Reporting Items for Systematic Reviews and Meta-Analyses (PRISMA) statement extension for scoping reviews (PRSIMA-ScR) [25]. Three separate searches were performed, each addressing a different aspect of the research question. A first search was conducted to identify relevant articles which assessed the nasal carriage of pathogens in people after exposure to infected animals. The inclusion criteria set for this search were (a) studies published in peer-reviewed journals in English or Dutch; (b) studies which were related to animal agriculture and/or veterinary medicine; and (c) studies which assessed the mechanical nasal carriage of pathogens through people after exposure to infected animals. Exclusion criteria were (a) studies in which nasal swabs were taken of animals only; (b) studies in which nasal swabs were not collected or not related to contact with infected animals; (c) studies focussed solely on non-production animals; (d) reviews, commentaries and other non-primary studies; and (e) studies in which humans served as biological hosts for infection rather than mechanical carriers. The used keywords and their strings were adjusted depending on the database used (Supplementary Table S2). The following database-specific search strings were used: PubMed: (“human” OR “people” OR “person *” OR “investigator” OR “veterinarian *” OR “veterinary” OR “worker *” OR “farmer *” OR “livestock worker *”) AND (“livestock” OR “poultry” OR “farm” OR “animal production” OR “cattle” OR “dairy animals” OR “dairy cattle” OR “beef production” OR “beef cattle” OR “buffalo *” OR “pig” OR “swine” OR “goat” OR “sheep” OR “chicken” OR “broiler” OR “layer” OR “turkey” OR “duck”) AND (“sick” OR “ill” OR “diseased” OR “infected” OR “confirmed infection” OR “disease outbreak *” OR “animal infection”) AND (“nasal” OR “nares” OR “nasopharyngeal” OR “nasal cavity” OR “upper respiratory tract”) AND (“carriage” OR “colonisation” OR “colonization” OR “carrier”); Web of Science: TS = ((“human *” OR “people *” OR “person *” OR “investigator *” OR “veterinarian *” OR “veterinary” OR “worker *” OR “farmer *” OR “livestock worker *”) AND (“livestock” OR “poultry” OR “farm” OR “animal production” OR “cattle” OR “dairy animal *” OR “dairy cattle” OR “beef production” OR “beef cattle” OR “buffalo *” OR “pig *” OR “swine *” OR “goat *” OR “sheep *” OR “chicken *” OR “broiler *” OR “layer *” OR “turkey *” OR “duck *”) AND (“sick” OR “ill” OR “diseased” OR “infected” OR “confirmed infection” OR “disease outbreak *” OR “animal infection”) AND (“nasal” OR “nares” OR “nasopharyngeal” OR “nasal cavity” OR “upper respiratory tract”) AND (“carriage” OR “colonisation” OR “colonization” OR “carrier”)); Scopus: TITLE-ABS-KEY ((“human *” OR “people *” OR “person *” OR “investigator *” OR “veterinarian *” OR “veterinary” OR “worker *” OR “farmer *” OR “livestock worker *”) AND (“livestock” OR “poultry” OR “farm” OR “animal production” OR “cattle” OR “dairy animal *” OR “dairy cattle” OR “beef production” OR “beef cattle” OR “buffalo *” OR “pig *” OR “swine *” OR “goat *” OR “sheep *” OR “chicken *” OR “broiler *” OR “layer *” OR “turkey *” OR “duck *”) AND (“sick” OR “ill” OR “diseased” OR “infected” OR “confirmed infection” OR “disease outbreak *” OR “animal infection”) AND (“nasal” OR “nares” OR “nasopharyngeal” OR “nasal cavity” OR “upper respiratory tract”) AND (“carriage” OR “colonisation” OR “colonization” OR “carrier”)).

A second search was conducted to identify studies which evaluated downtime as a biosecurity measure. Inclusion criteria set during the second search were (a) studies published in peer-reviewed journals in English or Dutch; (b) studies which were related to animal agriculture and/or veterinary medicine; and (c) studies which assessed downtime as a biosecurity measure to prevent indirect pathogen transmission through people. Exclusion criteria were (a) studies which maintained an alternative or incompatible definition of downtime; (b) studies which did not evaluate downtime as a biosecurity measure or did not report outcomes related to infection risk or transmission compliance; (c) studies focusing exclusively on non-production animals; and (d) reviews, commentaries and other non-primary studies. The used keywords and their strings: (“biosecurity” OR “farm biosecurity” OR “animal biosecurity” OR “preventive veterinary medicine” OR “herd health management”) AND (“livestock” OR “poultry” OR “farm” OR “animal production” OR “cattle” OR “dairy animals” OR “dairy cattle” OR “beef production” OR “beef cattle” OR “buffalo*” OR “pig” OR “swine” OR “goat” OR “sheep” OR “chicken” OR “broiler” OR “layer” OR “turkey” OR “duck”) AND (“downtime” OR “down time” OR “animal avoidance period” OR “animal free period” OR “pig free period” OR “pig avoidance period” OR “swine free period” OR “swine avoidance period” OR “poultry free period” OR “poultry avoidance period” OR “chicken free period” OR “chicken avoidance period” OR “broiler free period” OR “broiler avoidance period” OR “layer free period” OR “layer avoidance period” OR “duck free period” OR “duck avoidance period” OR “turkey free period” OR “turkey avoidance period” OR “sheep free period” OR “sheep avoidance period” OR “goat free period” OR “goat avoidance period” OR “cattle free period” OR “cattle avoidance period”). They were adjusted depending on the database used (Supplementary Table S3).

A third search was performed to increase the number of relevant articles used to answer the research question and focussed on the indirect transmission of pathogens through people. The inclusion criteria were similar to the second search and were broadened to include studies which assessed biosecurity measures aimed at preventing indirect pathogen transmission through people. The exclusion criteria were (a) studies that used a non-experimental or purely descriptive design that did not allow any assessment of the effect of a biosecurity measure on indirect transmission; (b) studies focussed exclusively on non-production animals; (c) reviews, commentaries and other non-primary studies; and (d) duplicated studies already included from the second literature search. For this search, the same search engines and search strings were used as in the second search, with the substitution of the ‘area of interest’ with the term “indirect transmission” (Supplementary Table S4).

For all searches performed, to increase the available literature, a cited reference search was also conducted for all included articles using the same three databases, as well as by manually screening the reference lists of included articles. Grey literature was systematically searched via Google Scholar, conference proceedings, and targeted sites (e.g., WOAH, EUR-Lex, the agricultural departments of national governments such as the United States Department of Agriculture, and industry organisations such as Red Tractor in the United Kingdom). No date or peer-review limits applied, but the inclusion and exclusion criteria mirrored the primary searches.

### 2.1. Data Extraction

Using the inclusion criteria, an initial screening of the articles was carried out by the first author. For a full-text analysis, the selected articles were moved to the Mendeley reference manager (Elsevier, Amsterdam, The Netherlands). Subsequently, data from the selected relevant articles were extracted and recorded in a Microsoft Excel worksheet (Microsoft Corp., Santa Rosa, CA, USA). The extracted data included in the worksheet were the country where the study was conducted, the year of the study, the type of study, the animal species, the farm type, the biosecurity measures evaluated, the pathogen investigated, the results of the evaluated biosecurity measures and the study’s citations.

### 2.2. Data Analysis

Analysis of the data from the included studies was mainly descriptive and focussed on factors such as livestock species, pathogens, study type and the effect of different studied biosecurity measures on indirect pathogen transmission through contaminated personnel and visitors.

### 2.3. Assessment of Study Quality and Limitations

No formal quality assessment tools were applied, consistent with a scoping review methodology. A narrative evaluation identified key limitations:

- Early studies by Sellers et al. used artificial conditions (prolonged coughing and sneezing into muzzles) without negative controls.
- Downtime trials were limited to two porcine studies with small sample sizes and short follow-ups.
- A heavy reliance on PCR detection in the nasal carriage detection studies, which overestimates viable virus compared to virus isolation techniques.
- Poultry and ruminant evidence is mostly observational (surveys and modelling), with no controlled downtime studies and only one indirect transmission study including sheep.
- Language restrictions to English and Dutch publications may have excluded relevant non-European studies.

These were noted during synthesis to contextualise findings.

## 3. Results

### 3.1. Study Characteristics

**Figure 1:**
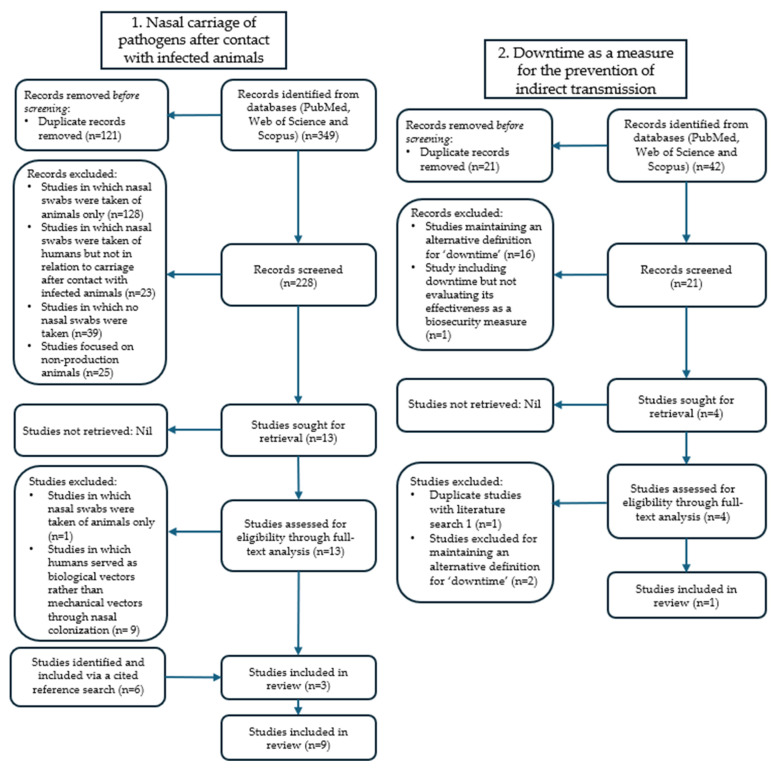

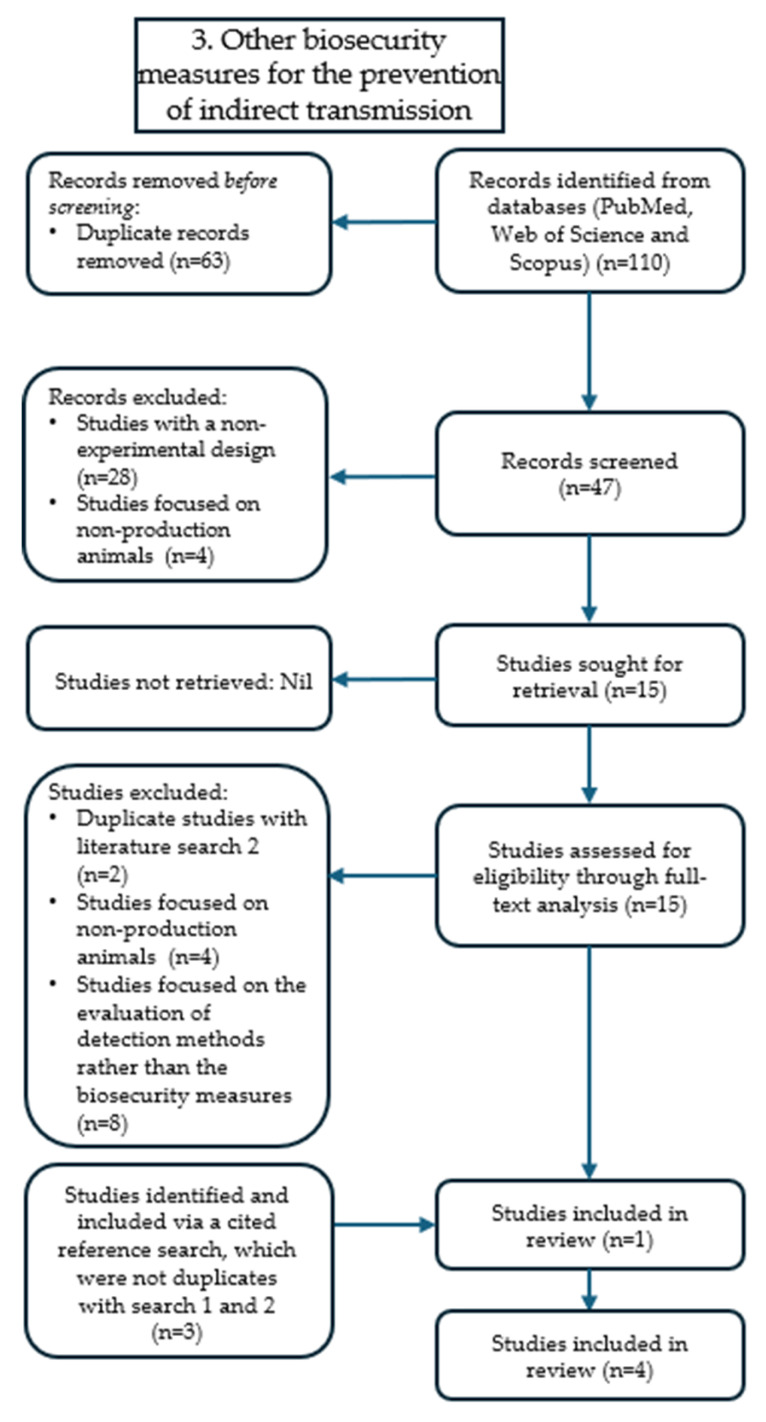
Flow chart of the first, second and third systematic literature searches conducted in line with the PRISMA-ScR procedure, aiming to answer the research question on the necessity of downtime as a biosecurity measure to prevent indirect pathogen transmission through personnel and visitors.

The primary search on “the nasal carriage of pathogens in people after exposure to infected animals” resulted in 349 articles, of which 121 were duplicates. The remaining 228 records were screened, of which 13 were selected for full-text analysis, and finally 3 were selected. An additional six articles were identified and selected through a cited reference search, resulting in a total of nine articles included through the first search (Figure 1). The second search on “downtime” resulted in 42 articles from the three databases. After the removal of duplicates, the abstracts of 21 articles were screened by applying the inclusion criteria, resulting in the selection of 4 articles for a full-text analysis. Of these four articles, two remained, of which one was already identified in the primary search, resulting in one unique article being included in the scoping review. The third literature search on the “indirect transmission of pathogens through people” initially resulted in 110 records, of which 47 remained after identifying and removing duplicates. For the full-text analysis 15 articles were selected, which resulted in 3 articles, of which 2 were excluded, as they overlapped with studies included from the first literature search, and 1 study remained to be included in the review (Figure 1). The cited reference search resulted in the inclusion of three more articles which were not duplicates of the results of the first or second searches. Finally, the combination of the three systematic literature searches (and the supplementary search techniques used) resulted in the inclusion of 14 unique peer-reviewed papers. An overview of all papers included, along with the search categories to which they contributed, can be found in Table 1.

### 3.2. Nasal Carriage of Pathogens After Exposure to Infected Animals

Nine studies examined nasal swabs from investigators after exposure to infected animals (Table 2). In the two foundational studies by Sellers et al. [2,3], FMDV was detected in all nasal swabs taken directly after exposure to experimentally infected pigs and sheep. In the first study, nasal swabs were taken up to 48 h after exposure, and the virus was detected in the nasal swabs of 8 out of 10 investigators between 2.5 and 4.5 h after exposure, despite investigators showering and changing clothing [2]. At 22–24 h, only 1 out of 10 swabs remained positive, which persisted at 28 h after exposure but not at 48 h. In the second study, investigators with positive nasal swabs proceeded to attempt to transfer the virus to naïve steers by coughing, sneezing, breathing and snorting into the muzzles of the animals for 2.5 min in total. One of four steers became infected 14 days later [3].

Amass et al. [21,26] also investigated FMDV nasal carriage. In the first study, the virus was detected in the nasal swab of one of the four investigators directly after showering, although it cleared within 12 h [26]. In the second study, the virus was not detected in any nasal swabs [21]. Despite virus detection by indirect enzyme-linked immunosorbent assay (ELISA), which detects live, infectious virus, no transmission occurred to sentinel pigs after showering and changing outerwear and boots or after hand hygiene and changing clothes and boots only. In contrast, sentinel sheep were infected when investigators performed only hand hygiene and changing of boots and clothes without showering. In a fifth study on FMD [27], polymerase chain reaction (PCR) detected virus in 36 out of 86 nasal swabs immediately after exposure to infected pigs, sheep and cattle, but virus isolation (VI) only detected viable virus in 3 out of the 36 PCR positive swabs. Within 16 to 22 h, all swabs were non-infective.

Other studies on different pathogens report mixed findings. Detection of *Mycoplasma hyopneumoniae* (*M. hyopneumoniae*) via PCR occurred in 15% (16/108) of farmers exposed to infected pigs [28], but viability could not be determined. Due to the cross-sectional study design, it was not possible to determine whether the positive PCRs of farmers were the cause or the result of the positive PCRs in the pigs, and it could not be determined if they formed a source of infection for the pigs. Two studies focussed on PRRSV; Otake et al. [29] reported no detection, whereas Amass et al. [30] detected PRRSV in the nasal swabs of one investigator with PCR 48 h after contact but not in earlier swabs of the same investigator, leading to doubts about sample contamination. No transmission to pigs was observed in either of the two studies. In ruminants, Oma et al. [31] found low levels of bovine coronavirus (BCoV) and bovine respiratory syncytial virus (BRSV) viral RNA in nasal mucosa for a maximum of 5 h after contact with infected calves in experimental settings. However, when infectivity was tested in the two swabs with the highest level of viral RNA per virus, the viruses collected were non-infective. In field outbreak conditions, nasal carriage was less common, with only 1 of 20 (5%) positive nasal swabs and only within 30 min after exposure [31].

Overall, seven of the nine studies detected nasal carriage after exposure to infected animals, but recovery of viable infectious virus was only described in three studies [2,26,27]. Only the study of Sellers et al. [3] demonstrated potential transmission to naïve animals under experimental conditions.

### 3.3. Downtime as a Measure to Prevent Mechanical Transmission

Two studies explicitly examined the effectiveness of downtime as a biosecurity measure in pig production [29,32]. For poultry and ruminant production, no publications on the effectiveness or necessity of downtime implementation were found.

In a study by Otake et al. [29] focussing on PRRSV, sentinel pigs became infected in two out of four replicates after direct contact with exposed investigators who applied no hygiene measures. In contrast, no transmission occurred when investigators implemented one of three protocols: they changed their coveralls and boots and washed their hands, they removed their coveralls and showered before donning fresh coveralls, or they removed their coveralls, showered and implemented a downtime period of 12 h before donning fresh coveralls. Pitkin et al. [32] built further on the previous study design and recorded the effects of a downtime period of one night (14–16 h), during which personnel showered and wore clean clothing, on the spread of PRRSV and *M. hyopneumoniae* over a close to four-year period. They found no pathogen transmission when these measures were taken. The inclusion of a spread control group, where investigators had direct contact with the sentinel animals without performing any biosecurity measures, showed indirect transmission of PRRSV to be possible, with all 10 pigs testing positive for PRRSV 10 days after contact. The inclusion of a downtime control group for PRRSV, where investigators showered and changed their clothing and boots but did not implement downtime, confirmed no additional benefit of downtime on top of these measures, as all sentinel animals in the control group remained negative throughout the duration of the study.

### 3.4. Other Biosecurity Measures to Prevent Mechanical Transmission

Indirect transmission of pathogens through contaminated clothing and boots (fomites) has been demonstrated to be possible for several dominant pathogens in the pig, ruminant and poultry industries such as PRRSV, porcine epidemic diarrhoea virus (PEDV), *M. hyopneumoniae*, *Escherichia coli* (*E. coli*), FMDV, paratuberculosis, BCoV and BRSV [21,29,32,33,34,35]. Consequently, biosecurity measures such as farm-specific or stable-specific clothing, the disinfection or changing of boots, hand hygiene and showering are implemented in varying degrees in different sectors.

Nine experimental studies assessed the efficacy of biosecurity measures directed at preventing the indirect transmission of pathogens through contaminated persons. Eight [21,29,30,32,36,37,38,39] out of these nine experimental studies focussed on pigs and one study included pigs and sheep in their set up [26]. No experimental studies were found for cattle and poultry.

#### 3.4.1. Hand Hygiene and Changing of Clothes and Boots

Seven of the nine studies had investigators perform clothing and boot changes together with hand hygiene before contact with sentinel animals. In five of these studies, investigating the transmission of FMDV, PRRSV, transmissible gastroenteritis virus (TGEV) and PEDV, pathogen transmission did not occur when these hygiene measures were implemented, in contrast to when these measures were not taken [21,26,29,36,39]. However, Amass et al. [26] found that sheep exposed to the same investigators became infected with FMDV despite these measures, while pigs did not. Another study found that, while these measures reduced the transmission of a porcine strain of enterotoxigenic *E. coli*, it did not fully prevent it [37]. Similarly, in the study by Allerson et al. [38], all sentinel pigs in one of two replicates were positive for Influenza A virus (IAV) after investigators implemented these measures, indicating that the effectiveness of these biosecurity measures varies for different pathogens and animal species.

#### 3.4.2. Showering and Changing of Clothes and Boots

Eight of the nine studies investigated showering in addition to changing clothing and footwear. The effectiveness of showering could not be evaluated for the study by Amass et al. [30] on PRRSV, as PRRSV was not transmitted to naïve control pigs when contaminated investigators had direct contact with the animals without performing any biosecurity measures. Nevertheless, in all cases [21,26,29,30,32,36,37,39], showering completely prevented pathogen transmission, regardless of the investigated pathogen and animal species. The only exception was the influenza study [38], which did not include a showering group. This evidence suggests that showering provides an added benefit over hand washing and changing of clothes and boots in specific cases, for *E. coli* in pigs and for FMDV in sheep, with the latter being the only pathogen studied in relation to small ruminants.

**Table 1:** Overview of all studies included in the review via the three literature searches, along with the categories to which they contribute and the pathogen and animal species targeted.

Reference	Category	Pathogen	Species
Sellers et al., 1970 [2]	Nasal carriage	Foot and mouth disease virus (FMDV)	Pigs, sheep and cattle
Sellers et al., 1971 [3]	Nasal carriage	FMDV	Pigs and cattle
Amass et al., 2000 [30]	Nasal carriage and indirect transmission	Porcine reproductive and respiratory syndrome virus (PRRSV)	Pigs
Amass et al., 2003b [26]	Nasal carriage and indirect transmission	FMDV	Pigs and sheep
Amass et al., 2004 [21]	Nasal carriage and indirect transmission	FMDV	Pigs
Wright et al., 2010 [27]	Nasal carriage	FMDV	Pigs, cattle and sheep
Nathues et al., 2012 [28]	Nasal carriage	*Mycoplasma hyopneumoniae* (*M. hyopneumoniae*)	Pigs
Oma et al., 2018 [31]	Nasal carriage	Bovine coronavirus (BCoV) and bovine respiratory syncytial virus (BRSV)	Cattle
Otake et al., 2002 [29]	Nasal carriage, downtime and indirect transmission	PRRSV	Pigs
Pitkin et al. 2010 [32]	Downtime and indirect transmission	PRRSV and *M. hyopneumoniae*	Pigs
Alvarez et al., 2001 [36]	Indirect transmission	Transmissible gastroenteritis virus (TGEV)	Pigs
Amass et al., 2003a [37]	Indirect transmission	Enterotoxigenic *Escherichia coli* (*E. coli*)	Pigs
Allerson et al., 2013 [38]	Indirect transmission	Influenza A virus	Pigs
Kim et al., 2017 [39]	Indirect transmission	Porcine epidemic diarrhoea virus (PEDV)	Pigs

**Table 2:** Overview of studies in which nasal swabs were taken from people after exposure to infected animals to test for the nasal carriage of pathogens.

Reference	Study Design	Pathogen	Strain	Animal Species	Detection Method	Sampling Period	Positive Results
Sellers et al., 1970 [2]	Experimental	Foot and mouth disease virus (FMDV)	O1 BFS 1860 O1 Swiss 1/66 O2 Brescia A5 Eystrup A22 Iraq 24/64 C Lebanon 3/69 C Noville	Pigs, sheep and cattle	Virus isolation (VI) and serum neutralisation test	Immediately after exposure (*n* = 10) Investigators showered and changed clothing and were sampled again: 2.5 to 4.5 h after exposure (*n* = 10) 22–24 h after exposure (*n* = 10) 28 h after exposure (*n* = 10) 48 h after exposure (*n* = unknown)	Immediately after exposure: virus detected in 10/10 samples After showering and changing of clothing: 2.5–4.5 h after exposure: 8/10 samples 22–24 h after exposure: 1/10 samples 28 h after exposure: 1/10 samples 48 h after exposure: no virus detected
Sellers et al., 1971 [3]	Experimental	FMDV	O1 BFS 1860 C Noville	Pigs and cattle	Not specified (NS)	After half of the infected pigs were examined (*n* = 10) After showering, changing of clothes and interaction (examination of animals while coughing, sneezing, snorting and breathing in the muzzles of sentinel steers) (*n* = 8)	Virus was detected in all nasal swabs taken at both sampling periods One out of four sentinel steers became FMD positive after interaction with investigators
Amass et al., 2000 [30]	Experimental	Porcine reproductive and respiratory syndrome virus (PRRSV)	P-129	Pigs	Reverse transcription polymerase chain reaction (RT-PCR)	Before exposure (*n* = 10) Immediately after exposure (*n* = 10) 4–10 h after exposure and every 24 h for 96 h (*n* = 10) Immediately after showering (*n* = 5)	1 person, 48 h after exposure *
Otake et al., 2002 [29]	Experimental	PRRSV	VR-2332	Pigs	Polymerase chain reaction (PCR), VI and swine bioassay	Before exposure (*n* = 4) Immediately after exposure (*n* = 4) Before contact with sentinel pigs without any hygiene measures (*n* = 1) After hand washing and changing of clothes (*n* = 1) After showering and changing of clothes (*n* = 1) After showering and a downtime of 12 h (*n* = 1)	None
Amass et al., 2003b [26]	Experimental	FMDV	O/UK/2001	Pigs and sheep	Indirect enzyme-linked immunosorbent assay (ELISA)	After showering-out (*n* = 4); Daily for 4 consecutive days (*n* = 4)	1 person, directly after showering
Amass et al., 2004 [21]	Experimental	FMDV	O/TAW/1997	Pigs	Indirect ELISA	After showering-out (*n* = 4) Daily for 4 consecutive days (*n* = 4)	None
Wright et al., 2010 [27]	Cross-sectional	FMDV	Asia 1 HKN 5/05 O UKG 34/2001 O BFS 1860/67	Pigs, cattle and sheep	RT-PCR and VI	Before exposure (*n* = 10) Immediately after exposure (*n* = 86) 16 to 22 h after exposure (and showering) (*n* = 68)	Immediately after exposure: 35/86 PCR-positive 3/86 VI positive Between 16 and 22 h after exposure: 1/68 PCR-positive 0/68 VI positive
Nathues et al., 2012 [28]	Cross-sectional	*Mycoplasma* *hyopneumoniae*	NS	Pigs	RT-PCR	Within 24 h of last exposure (*n* = 108)	16/108 farmers positive
Oma et al., 2018 [31]	Experimental	Bovine coronavirus (BCoV) and bovine respiratory syncytial virus (BRSV)	BRSV O-4/N-11	Cattle	Integrated cell culture reverse transcription quantitative polymerase chain reaction (RT-qPCR)	BCoV experiment: before exposure; 0.5, 2, 4 and 6 h after exposure (*n* = 16) BRSV experiment: 0.5 h after exposure (*n* = 12) Winter dysentery outbreak: 0.5, 2, 4 h after exposure (*n* = 19)	BCoV experiment: after exposure: 0.5 h: 37/80 2 h: 10/68 4 h: 2/38 BRSV experiment: 0.5 h after exposure: 9/25 Winter dysentery outbreak: 0.5 h after exposure: 1/7

* Detected in a person where previous swabs were negative.

## 4. Discussion

### 4.1. Nasal Carriage and Downtime

Early studies by Sellers et al. [2,3] demonstrated nasal carriage of FMD virus and described the possibility of subsequent transmission to a naïve animal. However, these experiments relied on extraordinary measures to transmit the virus to naïve animals and did not include negative control groups of animals not exposed to contaminated investigators. Thus, it could not be confirmed that the observed transmission was truly due to virus exposure from investigators with nasal virus carriage. When more recent investigations [21,26] replicated such conditions under more natural interactions between investigators and animals, no consistent evidence of transmission through human nasal carriage was found. Although an infectious virus was detected in one investigator, transmission to pigs was prevented by basic hygiene measures of hand hygiene and changing of clothes and boots. However, transmission was observed in sheep despite these hygiene measures, but since the same investigator did not transmit to sheep after showering, this suggests alternative transmission routes rather than nasal carriage. Later studies demonstrated that PCR detection often overestimates infective virus [27,31], which could justify why PCR positive nasal swabs in other studies did not result in virus transmission. Considering that all experimental and field studies conducted after those by Sellers et al. [3] have consistently failed to reproduce the risk of human nasal carriage, the impact of the 1971 study on downtime implementation and the perceived risk of human nasal carriage seems disproportionate. At the same time, the repeated finding of PCR positive samples indicates that exposed individuals can carry viral material on the upper respiratory and facial region. Nasal detection, whether or not a viable virus is present, can therefore be interpreted as an indicator for contamination on adjacent skin, hair and items that contact the face (e.g., hands, gloves and masks), and is thus more appropriately situated within the broader fomite transmission pathway than as evidence of transmission via the nasal respiratory route.

Only two experimental studies were found that directly evaluated downtime [29,32], both focussing on pigs. Neither demonstrated an added benefit of downtime once basic biosecurity measures, such as hand hygiene, changing of clothing and boots, or showering, were applied. The inclusion of a downtime control group in the Pitkin et al. [32] study where investigators showered but did not implement a downtime period suggests that any effect seen during downtime is due to the hygiene measures taken within this period rather than the 24–72 h period itself. Pitkin et al. [32] even concluded that any time spent on downtime was effectively wasted, under the specific conditions of their study, further supporting a shift in emphasis from the duration of downtime towards the consistent application of effective hygiene measures.

### 4.2. Other Hygiene-Based Biosecurity Measures

While no clear evidence was found supporting downtime as an effective and necessary biosecurity measure, the review of eight pathogens demonstrated that hygiene-based measures of hand cleaning with clothing and boot changes were consistently effective at preventing indirect mechanical transmission. Showering offered additional value only in a minority of cases, with *E. coli* in pigs and FMD virus in sheep [26,37]. Its greater effectiveness in these scenarios is likely related to the risk that contamination could extend beyond hands and clothing to hair and facial areas, as has been documented with respiratory pathogens such as PEDV [39]. The evidence suggests that the incremental benefit of showering varies with pathogen characteristics, the degree of animal contact and herd health status. For high-risk pathogens such as PEDV or African swine fever, for individuals with frequent animal contact, or when entering high-health herds, showering remains a valuable, evidence-supported layer of protection. These findings emphasise a risk-based approach to biosecurity, where rigorous hygiene practices form the foundation and additional measures, such as showering, are implemented according to the specific level of infection risk.

### 4.3. Discrepancy Between Scientific Evidence and Policy Coverage

This scoping review identifies a major gap between the widespread policy use of downtime as a biosecurity measure and the extremely limited scientific evidence directly assessing its effectiveness. To date, only two controlled experimental studies directly evaluating downtime have been identified, both of which were conducted on pigs, providing a narrow and species-specific evidence base. Overall, research on other biosecurity measures related to indirect transmission is largely focussed on pigs, and data for poultry and cattle are mostly based on surveys and modelling, with no experimental assessments of downtime or indirect pathogen transmission for key pathogens in these species. This further highlights the gap between the evidence and policy, considering the widespread implementation of downtime even in species for which the measure has never even been investigated.

Despite this narrow evidence base, requirements for downtime are widespread in policies across multiple production systems and species. This is especially concerning given that downtime in policy is often related to disease outbreaks, yet it may divert attention away from proven biosecurity measures and leave critical transmission routes insufficiently controlled. Compliance is often difficult to verify, and visitors may not fully adhere to requirements such as changing clothes, showering, or disinfecting equipment during the downtime period. Moreover, pathogens such as BCoV and BRSV can persist on fomites beyond 24 h, long after their detection on nasal swabs [31], and other pathogens such as *Salmonella* spp. may survive in manure for several weeks to a month [40], making a 24 h downtime insufficient to block transmission if the basic hygiene measures of changing clothes and footwear and washing hands are not applied. Furthermore, the practical implications of downtime on professional visitors such as veterinarians and catching crews do not align with regular work in these sectors.

Beyond its originally intended role of reducing the risk associated with human nasal carriage, downtime also appears to serve practical and psychological functions that likely contribute to its institutionalisation in biosecurity policies. It can discourage unnecessary farm visits and act as an administrative tool to structure and space visits, while the notion of a mandatory pause between farms may heighten the awareness of cross-farm contamination risks and create a conscious break in the visitation chain. These non-trivial management benefits do not, however, constitute evidence that downtime is biologically effective as a primary measure to prevent pathogen transmission, since observed effects are confounded by hygiene measures performed during the avoidance period and remain unverifiable in practice.

If downtime were to maintain a role in the external biosecurity of farms, its role is better defined as a supplementary administrative control to manage visit frequency and scheduling, but always subordinate to verifiable hygiene protocols. Emphasis should be placed on evidence-based biosecurity practices, including hygiene locks, changing into farm-specific clothing and footwear, disinfection of equipment, facility zoning, and restricting visits to essential personnel. These measures not only have stronger evidence but can also be implemented and monitored more reliably on a day-to-day basis, thus not only mitigating outbreaks but also preventing them.

### 4.4. Humans as Biological Vectors

The ability of humans to act as biological vectors for the transmission of diseases to animals presents another challenge to the biosecurity of farms, which cannot be solved by a downtime of 24–72 h on average. Outbreaks such as the 2009 influenza pandemic, during which the introduction of the virus from infected humans to swine was more frequent than the zoonotic transmission from swine to humans [41], illustrate that human-to-animal transmission can occur, despite the implementation of hygiene measures. In one case, a 36 h downtime period in addition to hygiene measures was still unsuccessful in preventing pathogen introduction into the herd [42]. Other pathogens for which humans can serve as biological vectors when they themselves are carriers include tuberculosis [43], SARS-Coronavirus 2 [44,45,46] and Methicillin-resistant Staphylococcus aureus (MRSA) [47]. In this context, a relevant downtime period would be the pathogen-specific duration of illness or shedding. A generic downtime of 24–72 h is ineffective in preventing this transmission route and protecting animals from humans serving as biological vectors. Evidence-based strategies which are effective against human carriers of pathogens include restricting farm access for symptomatic personnel, enforcing appropriate sick-leave policies, providing health screenings for pathogens for which humans could be asymptomatic carriers, such as tuberculosis and MRSA, and where relevant, vaccination and use of personal protective equipment (e.g., respiratory masks) [48,49,50].

## 5. Conclusions

Based on the available literature, there is no clear evidence supporting downtime, nor any indication that it provides additional benefit over other hygiene-based biosecurity measures. Although nasal carriage of pathogens has been detected, only one experimental study reported transmission to naïve animals, creating further doubt on the necessity of downtime. Given the pig-focussed evidence and absence of data for poultry and ruminants, conclusions remain species- and context-specific. Future research should prioritise experimental studies on key avian and ruminant pathogens, cost–benefit analyses of downtime versus hygiene infrastructure, and behavioural compliance studies. Emphasis in policy and practice must be placed on practical and evidence-based biosecurity measures which effectively prevent indirect pathogen transmission, such as hand hygiene, clothing and boot changes and, if necessary, showering.

## Data Availability

No new data were created or analysed in this study. Data sharing is not applicable to this article. All data supporting the findings are from previously published studies, which have been appropriately cited.

## Supplementary Materials

The following supporting information can be downloaded at https://www.mdpi.com/article/10.3390/ani16020205/s1: Table S1: A non-exhaustive overview of legislation and industry standards including downtime as a biosecurity measure. Table S2: Search approach focussed on the mechanical nasal carriage of pathogens through people after exposure to infected animals. Seach strategy applied in PubMed. Table S3: Search approach focussed on downtime as a biosecurity measure to prevent the indirect transmission of pathogens through people. Search strategy applied in PubMed. Table S4: Search approach focussed on biosecurity measures to prevent the indirect transmission of pathogens through people. Search strategy applied in PubMed [51,52,53,54,55,56].

## Abbreviations

The following abbreviations are used in this manuscript:

FMD	Foot and mouth disease
EU	European Union
FMDV	Foot and mouth disease virus
PRRSV	Porcine reproductive and respiratory syndrome virus
AI	Avian influenza
WOAH	World Organisation for Animal Health
PRISMA	Preferred Reporting Items for Systematic Reviews and Meta-Analyses
PRIMSA-ScR	Preferred Reporting Items for Systematic Reviews and Meta-Analyses extended statement for scoping reviews
VI	Virus isolation
ELISA	Indirect enzyme-linked immunosorbent assay
PCR	Polymerase chain reaction
RT-PCR	Reverse transcription polymerase chain reaction
BCoV	Bovine coronavirus
BRSV	Bovine respiratory syncytial virus
*M. hyopneumoniae*	*Mycoplasma hyopneumoniae*
PEDV	Porcine endemic diarrhoea virus
TGEV	Transmissible gastroenteritis virus
*E. coli*	*Escherichia coli*
IAV	Influenza A virus
MRSA	Methicillin-resistant Staphylococcus aureus
