# Supplementary material for: Are Visitor and Personnel Downtime Restrictions an Effective Biosecurity Measure to Prevent the Indirect Transmission of Pathogens to Livestock?
Source: Animals (Basel). 2026 Jan 9;16(2):205. doi: 10.3390/ani16020205 (PMC12837341; doi:10.3390/ani16020205)
Supplement: Supplementary file 1 [file animals-16-00205-s001.zip › Supplementary materials - Downtime manuscript.pdf]

Supplementary table S1**:** A non-exhaustive overview of legislation and industry standards including downtime as a biosecurity measure

Organisation	Legislation/Standard	Geographical area of implementation	Relevant species	Relevant pathogen	Statement	Reference
**World Organisation for Animal Health**	Terrestrial Animal Health Code; Article 6.5.5	Global	Poultry	*Not specified (NS)*	*"Personnel and visitors should not have had recent contact with other poultry, poultry waste, or poultry processing plant(s). This time period should be based on the level of risk of transmission of infectious agents. This will depend on the poultry production purpose, biosecurity procedures and infection status"*	[14]
**European Commission**	Commission regulation 2021/934	European Union	Pigs	Classical swine fever	*"Persons entering into contact with kept porcine animals on the establishment must not have engaged in any hunting activity related to wild porcine animals or have had any other contact with wild porcine animals for a period of at least 48 hours before entering into the establishment"*	[16]
**European Commission**	Commission regulation 2023/594	European Union	Pigs	African swine fever	*"the absence of any contact with kept porcine animals for a period of at least 48 hours from the time of ending of any hunting activity related to wild porcine animals or any other contact with wild porcine animals"*	[51]
**European Food Safety Authority**	Scientific Opinion on Avian Influenza	European Union	Poultry	Avian Influenza	*"Persons can only enter the holding if they had no contact with poultry, poultry waste*	[15]

					and/or a poultry processing material at another holding (including backyard) within the previous 24 h. This time could be enlarged in a high-risk situation, e.g. depending on the production system, virus characteristics, contact with wild birds (e.g. bird ringing or hunting), etc."	
**European Feed Manufacturers Federation**	Recommendations for the Development of a Biosecurity Plan in the EU Compound Feed Industry	European Union	*NS*	*NS*	*"People should not be allowed to enter into a livestock holding sensitive to a pathogen within a predefined period of time following contacts with feral or domestic animals carrying potentially this pathogen"*	[52]
**United States Department of Agriculture**	Biosecurity checklist as part of the 'Defend the flock' campaign	United States of America	Poultry	*NS*	*"Always ask visitors if they've been in contact with wild birds or pet birds or if they have their own poultry. If so, they should not go near your flock. Some avian disease agents can survive in a person's nose, without actually infecting the person, for 2 days."*	[53]
**United States Department of Agriculture**	Manual on Protecting America From Foot-and-Mouth Disease and Other High-Consequence Livestock Diseases	United States of America	*NS*	Foot-and-mouth disease and other high-consequence livestock diseases	*"If you visited a farm or had any contact with livestock on your trip, avoid all contact with livestock, zoo animals, or wildlife for 5 days after your return to the United States."*	[17]

**Australian Department of Agriculture, Fisheries and Forestry**	Advice to travellers (entering Australia)	Australia	Cloven-hoofed animals (including cattle, sheep, goats and pigs)	Foot-and-mouth disease	*"Declare on your Incoming Passenger Card if you have visited a rural area or been in contact with, or near, farm animals."*<br><br>*"Avoid farms and livestock for the first seven days after arriving in Australia"*	[18]
**Animal Health Australia**	National Farm Biosecurity Manual for Pork Production	Australia	Pigs	*NS*	*"There is a written protocol that details piggery entry requirements for people who have recently been overseas. If there has been any contact with cloven-hoofed animals whilst outside Australia, international entries should have at least 48 hours of no animal contact before arriving on-farm"*	[54]
**Netherlands Food and Consumer Product Safety Authority**	Hygiene protocol for poultry farm visitors	The Netherlands	Poultry	Avian Influenza	*"For visits in the context of:*<br>*- conducting scientific research.*<br>*- control of the establishment with (commercially kept) poultry due to participation in a quality system.*<br>*- assurance of central antibiotic registration.*<br>*- supervision of flocks of commercial poultry.*<br>*the visitor must not have visited any other establishment with commercially-reared birds in the 24 hours prior to the visit and the visitor can demonstrate this for control purposes."*	[20]

**The Federal Agency for the Safety of the Food Chain**	Instructions for farm veterinarians on the risk survey for pig farms	Belgium	Pigs	*NS*	*"The farmer must prohibit contact with pigs from his farm for anyone who has had direct contact with feral pigs during the previous 72 hours."*<br><br>*"The livestock farmer must prohibit entry to his farm of any vehicle, person and any material that/which in the previous 72 hours:*<br>*- 1° has been in contact with pigs from a third country or an area at risk originating from pigs, or*<br>*- 2° has been on a holding or in a place in a third country or in a risk area, where pigs are kept"*	[55]
**Red Tractor**	Chicken Standards	United Kingdom	Poultry	*NS*	*"A record is kept of all visitors who are not site specific staff members, detailing:*<br>*- site name of last contact with poultry within the last 7 days (or within 14 days if visited overseas)*<br>*– confirmation of no vomiting or diarrhoea, or flu like symptoms in the last 24 hours"*<br><br>*"Entry is refused if there is significant risk to the health and welfare of the birds"*	[56]

Supplementary Tables S2 to S4: Search approaches for scoping review for the research question "'Is downtime an effective biosecurity measure to prevent the indirect transmission of pathogens through people?": search strategy applied in PubMed

Supplementary Table S2: Search approach focussed on the mechanical nasal carriage of pathogens through people after exposure to infected animals

Pubmed:

Population	
1	"human"
2	"people"
3	"person*"
4	"investigator*"
5	"veterinarian*"
6	"veterinary"
7	"worker*"
8	"farmer*"
9	"livestock worker*"
10	("human" OR "people" OR "person*" OR "investigator" OR "veterinarian*" OR "veterinary" OR "worker*" OR "farmer*" OR "livestock worker*")
11	OR/1-9
Context	
12	"livestock"
13	"poultry"
14	"cattle"
15	"dairy animals"
16	"dairy cattle"
17	"beef production"
18	"beef cattle"
19	"buffaloes"
20	"pig"
21	"swine"
22	"goat"
23	"sheep"
24	"chicken"
25	"broiler"
26	"layer"
27	"turkey"

28	"duck"
29	"sick"
30	"ill"
31	"diseased"
32	"infected"
33	"confirmed infection"
34	"disease outbreak*"
35	"animal infection"
36	("livestock" OR "poultry" OR "farm" OR "animal production" OR "cattle" OR "dairy animals" OR "dairy cattle" OR "beef production" OR "beef cattle" OR "buffalo*" OR "pig" OR "swine" OR "goat" OR "sheep" OR "chicken" OR "broiler" OR "layer" OR "turkey" OR "duck") AND ("sick" OR "ill" OR "diseased" OR "infected" OR "confirmed infection" OR "disease outbreak*" OR "animal infection")
37	(OR/12-28) AND (OR/29-35)
Area of Interest	
38	"nasal"
39	"nares"
40	"nasopharyngeal"
41	"nasal cavity"
42	"upper respiratory tract"
43	"carriage"
44	"colonisation"
45	"colonization"
46	"carrier"
47	("nasal" OR "nares" OR "nasopharyngeal" OR "nasal cavity" OR "upper respiratory tract") AND ("carriage" OR "colonisation" OR "colonization" OR "carrier")
48	(OR/38-42) AND (OR/43-46)
Outcomes	
49	("human" OR "people" OR "person*" OR "investigator" OR "veterinarian*" OR "veterinary" OR "worker*" OR "farmer*" OR "livestock worker*")
50	("livestock" OR "poultry" OR "farm" OR "animal production" OR "cattle" OR "dairy animals" OR "dairy cattle" OR "beef production" OR "beef cattle" OR "buffalo*" OR "pig" OR "swine" OR "goat" OR "sheep" OR "chicken" OR "broiler" OR "layer" OR "turkey" OR "duck") AND ("sick" OR "ill" OR "diseased" OR "infected" OR "confirmed infection" OR "disease outbreak*" OR "animal infection")
51	("nasal" OR "nares" OR "nasopharyngeal" OR "nasal cavity" OR "upper respiratory tract") AND ("carriage" OR "colonisation" OR "colonization" OR "carrier")

52	(“human” OR “people” OR “person*” OR “investigator” OR “veterinarian*” OR “veterinary” OR “worker*” OR “farmer*” OR “livestock worker*”) AND ("livestock" OR "poultry" OR "farm" OR "animal production" OR "cattle" OR "dairy animals" OR "dairy cattle" OR "beef production" OR "beef cattle" OR "buffalo*" OR "pig" OR "swine" OR "goat" OR "sheep" OR "chicken" OR "broiler" OR "layer" OR "turkey" OR "duck") AND (“sick” OR “ill” OR “diseased” OR “infected” OR “confirmed infection” OR “disease outbreak*” OR “animal infection”) AND (“nasal” OR “nares” OR “nasopharyngeal” OR “nasal cavity” OR “upper respiratory tract”) AND (“carriage” OR “colonisation” OR “colonization” OR “carrier”)
53	AND/ 10, 36, 47

Web of Science: TS=(("human*" OR "people*" OR "person*" OR "investigator*" OR "veterinarian*" OR "veterinary" OR "worker*" OR "farmer*" OR "livestock worker*") AND ("livestock" OR "poultry" OR "farm" OR "animal production" OR "cattle" OR "dairy animal*" OR "dairy cattle" OR "beef production" OR "beef cattle" OR "buffalo*" OR "pig*" OR "swine*" OR "goat*" OR "sheep*" OR "chicken*" OR "broiler*" OR "layer*" OR "turkey*" OR "duck*") AND ("sick" OR "ill" OR "diseased" OR "infected" OR "confirmed infection" OR "disease outbreak*" OR "animal infection") AND ("nasal" OR "nares" OR "nasopharyngeal" OR "nasal cavity" OR "upper respiratory tract") AND ("carriage" OR "colonisation" OR "colonization" OR "carrier"))

Scopus: TITLE-ABS-KEY(("human*" OR "people*" OR "person*" OR "investigator*" OR "veterinarian*" OR "veterinary" OR "worker*" OR "farmer*" OR "livestock worker*") AND ("livestock" OR "poultry" OR "farm" OR "animal production" OR "cattle" OR "dairy animal*" OR "dairy cattle" OR "beef production" OR "beef cattle" OR "buffalo*" OR "pig*" OR "swine*" OR "goat*" OR "sheep*" OR "chicken*" OR "broiler*" OR "layer*" OR "turkey*" OR "duck*") AND ("sick" OR "ill" OR "diseased" OR "infected" OR "confirmed infection" OR "disease outbreak*" OR "animal infection") AND ("nasal" OR "nares" OR "nasopharyngeal" OR "nasal cavity" OR "upper respiratory tract") AND ("carriage" OR "colonisation" OR "colonization" OR "carrier"))

Supplementary table S3: Search approach focussed on downtime as a biosecurity measure to prevent the indirect transmission of pathogens through people

Pubmed:

Population	
1	“livestock”
2	“poultry”
3	“cattle”
4	“dairy animals”
5	“dairy cattle”
6	“beef production”

7	“beef cattle”
8	“buffaloes”
9	“pig”
10	“swine”
11	“goat”
12	“sheep”
13	“chicken”
14	“broiler”
15	“layer”
16	“turkey”
17	“duck”
18	("livestock" OR "poultry" OR "farm" OR "animal production" OR "cattle" OR "dairy animals" OR "dairy cattle" OR "beef production" OR "beef cattle" OR "buffalo*" OR "pig" OR "swine" OR "goat" OR "sheep" OR "chicken" OR "broiler" OR "layer" OR "turkey" OR "duck")
19	OR/1-17
Area of Interest	
20	“downtime”
21	“down time”
22	“animal avoidance period”
23	“animal free period”
24	“pig free period”
25	“pig avoidance period”
26	“swine free period”
27	“swine avoidance period”
28	“poultry free period”
29	“poultry avoidance period”
30	“chicken free period”
31	“chicken avoidance period”
32	“broiler free period”
33	“broiler avoidance period”
34	“layer free period”
35	“layer avoidance period”
36	“duck free period”
37	“duck avoidance period”
38	“turkey free period”
39	“turkey avoidance period”

40	“sheep free period”
41	“sheep avoidance period”
42	“goat free period”
43	“goat avoidance period”
44	“cattle free period”
45	“cattle avoidance period”
46	(“downtime” OR “down time” OR “animal avoidance period” OR “animal free period” OR “pig free period” OR “pig avoidance period” OR “swine free period” OR “swine avoidance period” OR “poultry free period” OR “poultry avoidance period” OR “chicken free period” OR “chicken avoidance period” OR “broiler free period” OR “broiler avoidance period” OR “layer free period” OR “layer avoidance period” OR “duck free period” OR “duck avoidance period” OR “turkey free period” OR “turkey avoidance period” OR “sheep free period” OR “sheep avoidance period” OR “goat free period” OR “goat avoidance period” OR “cattle free period” OR “cattle avoidance period”)
47	OR/ 20-45
Context	
48	“biosecurity”
49	“farm biosecurity”
50	“animal biosecurity”
51	“preventive veterinary medicine”
52	“herd health management”
53	(“biosecurity” OR "farm biosecurity" OR "animal biosecurity" OR "preventive veterinary medicine" OR "herd health management")
54	OR/48-52
Outcomes	
55	("biosecurity” OR “farm biosecurity" OR "animal biosecurity" OR "preventive veterinary medicine" OR "herd health management")
56	("livestock" OR "poultry" OR "farm" OR "animal production" OR "cattle" OR "dairy animals" OR "dairy cattle" OR "beef production" OR "beef cattle" OR "buffalo*" OR "pig" OR "swine" OR "goat" OR "sheep" OR "chicken" OR "broiler" OR "layer" OR "turkey" OR "duck")
57	(“downtime” OR “animal avoidance period” OR “animal free period” OR “pig free period” OR “pig avoidance period” OR “swine free period” OR “swine avoidance period” OR “poultry free period” OR “poultry avoidance period” OR “chicken free period” OR “chicken avoidance period” OR “broiler free period” OR “broiler avoidance period” OR “layer free period” OR “layer avoidance period” OR “duck free period” OR “duck avoidance period” OR “turkey free period”

	OR “turkey avoidance period” OR “sheep free period” OR “sheep avoidance period” OR “goat free period” OR “goat avoidance period” OR “cattle free period” OR “cattle avoidance period”)
58	("biosecurity” OR “farm biosecurity" OR "animal biosecurity" OR "preventive veterinary medicine" OR "herd health management") AND ("livestock" OR "poultry" OR "farm" OR "animal production" OR "cattle" OR "dairy animals" OR "dairy cattle" OR "beef production" OR "beef cattle" OR "buffalo*" OR "pig" OR "swine" OR "goat" OR "sheep" OR "chicken" OR "broiler" OR "layer" OR "turkey" OR "duck") AND (“downtime” OR “down time” OR “animal avoidance period” OR “animal free period” OR “pig free period” OR “pig avoidance period” OR “swine free period” OR “swine avoidance period” OR “poultry free period” OR “poultry avoidance period” OR “chicken free period” OR “chicken avoidance period” OR “broiler free period” OR “broiler avoidance period” OR “layer free period” OR “layer avoidance period” OR “duck free period” OR “duck avoidance period” OR “turkey free period” OR “turkey avoidance period” OR “sheep free period” OR “sheep avoidance period” OR “goat free period” OR “goat avoidance period” OR “cattle free period” OR “cattle avoidance period”)
59	AND/ 18, 47, 53

Web of Science (TS= Topic field): TS=("livestock" OR "poultry" OR "farm" OR "animal production" OR "cattle" OR "dairy animals" OR "dairy cattle" OR "beef production" OR "beef cattle" OR "buffalo*" OR "pig" OR "swine" OR "goat" OR "sheep" OR "chicken" OR "broiler" OR "layer" OR "turkey" OR "duck") AND ("downtime" OR "down time" OR "animal avoidance period" OR "animal free period" OR "pig free period" OR "pig avoidance period" OR "swine free period" OR "swine avoidance period" OR "poultry free period" OR "poultry avoidance period" OR "chicken free period" OR "chicken avoidance period" OR "broiler free period" OR "broiler avoidance period" OR "layer free period" OR "layer avoidance period" OR "duck free period" OR "duck avoidance period" OR "turkey free period" OR "turkey avoidance period" OR "sheep free period" OR "sheep avoidance period" OR "goat free period" OR "goat avoidance period" OR "cattle free period" OR "cattle avoidance period") AND ("biosecurity" OR "farm biosecurity" OR "animal biosecurity" OR "preventive veterinary medicine" OR "herd health management")

Scopus (TITLE-ABS-KEY): TITLE-ABS-KEY(("livestock" OR "poultry" OR "farm" OR "animal production" OR "cattle" OR "dairy animals" OR "dairy cattle" OR "beef production" OR "beef cattle" OR "buffalo*" OR "pig" OR "swine" OR "goat" OR "sheep" OR "chicken" OR "broiler" OR "layer" OR "turkey" OR "duck") AND ("downtime" OR "down time" OR "animal avoidance period" OR "animal free period" OR "pig free period" OR "pig avoidance period" OR "swine free period" OR "swine avoidance period" OR "poultry free period" OR "poultry avoidance period" OR "chicken free period" OR "chicken avoidance period" OR "broiler free period" OR "broiler avoidance period" OR "layer free period" OR "layer avoidance period" OR "duck free period" OR "duck avoidance period" OR "turkey free period" OR "turkey avoidance period" OR "sheep free period" OR "sheep avoidance period" OR "goat free period" OR "goat avoidance period" OR "cattle free period" OR "cattle avoidance period") AND ("biosecurity"

OR "farm biosecurity" OR "animal biosecurity" OR "preventive veterinary medicine" OR "herd health management"))

Supplementary table S4: Search approach focussed on biosecurity measures to prevent the indirect transmission of pathogens through people

PubMed:

Population	
1	“livestock”
2	“poultry”
3	“cattle”
4	“dairy animals”
5	“dairy cattle”
6	“beef production”
7	“beef cattle”
8	“buffaloes”
9	“pig”
10	“swine”
11	“goat”
12	“sheep”
13	“chicken”
14	“broiler”
15	“layer”
16	“turkey”
17	“duck”
18	("livestock" OR "poultry" OR "farm" OR "animal production" OR "cattle" OR "dairy animals" OR "dairy cattle" OR "beef production" OR "beef cattle" OR "buffalo*" OR "pig" OR "swine" OR "goat" OR "sheep" OR "chicken" OR "broiler" OR "layer" OR "turkey" OR "duck")
19	OR/1-17
Area of Interest	
20	“indirect transmission”
22	(“indirect transmission”)
23	OR/22
Context	
24	“biosecurity”
25	“farm biosecurity”
26	“animal biosecurity”

27	“preventive veterinary medicine”
28	“herd health management”
29	(“biosecurity” OR "farm biosecurity" OR "animal biosecurity" OR "preventive veterinary medicine" OR "herd health management")
30	OR/23-27
Outcomes	
31	("biosecurity” OR “farm biosecurity" OR "animal biosecurity" OR "preventive veterinary medicine" OR "herd health management")
32	("livestock" OR "poultry" OR "farm" OR "animal production" OR "cattle" OR "dairy animals" OR "dairy cattle" OR "beef production" OR "beef cattle" OR "buffalo*" OR "pig" OR "swine" OR "goat" OR "sheep" OR "chicken" OR "broiler" OR "layer" OR "turkey" OR "duck")
33	(“indirect transmission”)
34	("biosecurity” OR “farm biosecurity" OR "animal biosecurity" OR "preventive veterinary medicine" OR "herd health management") AND ("livestock" OR "poultry" OR "farm" OR "animal production" OR "cattle" OR "dairy animals" OR "dairy cattle" OR "beef production" OR "beef cattle" OR "buffalo*" OR "pig" OR "swine" OR "goat" OR "sheep" OR "chicken" OR "broiler" OR "layer" OR "turkey" OR "duck") AND (“indirect transmission”)
35	AND/ 18, 23, 29

Web of Science (TS= Topic Field): TS=("livestock" OR "poultry" OR "farm" OR "animal production" OR "cattle" OR "dairy animals" OR "dairy cattle" OR "beef production" OR "beef cattle" OR "buffalo*" OR "pig" OR "swine" OR "goat" OR "sheep" OR "chicken" OR "broiler" OR "layer" OR "turkey" OR "duck") AND ("indirect transmission") AND ("biosecurity" OR "farm biosecurity" OR "animal biosecurity" OR "preventive veterinary medicine" OR "herd health management")

Scopus (TITLE-ABS-KEY): TITLE-ABS-KEY(("livestock" OR "poultry" OR "farm" OR "animal production" OR "cattle" OR "dairy animals" OR "dairy cattle" OR "beef production" OR "beef cattle" OR "buffalo*" OR "pig" OR "swine" OR "goat" OR "sheep" OR "chicken" OR "broiler" OR "layer" OR "turkey" OR "duck") AND ("indirect transmission") AND ("biosecurity" OR "farm biosecurity" OR "animal biosecurity" OR "preventive veterinary medicine" OR "herd health management"))
